# Early and Late Onset Side Effects of Photodynamic Therapy

**DOI:** 10.3390/biomedicines6010012

**Published:** 2018-01-29

**Authors:** Francesco Borgia, Roberta Giuffrida, Emanuela Caradonna, Mario Vaccaro, Fabrizio Guarneri, Serafinella P. Cannavò

**Affiliations:** Section of Dermatology—Department of Clinical and Experimental Medicine, University of Messina, 98125 Messina, Italy; robgiuffrida@unime.it (R.G.); emanuela.caradonna79@gmail.com (E.C.); mario.vaccaro@unime.it (M.V.); f.guarneri@tiscali.it (F.G.); patrizia.cannavo@unime.it (S.P.C.)

**Keywords:** non-melanoma skin cancers, photodynamic therapy, adverse events, pain, erythema, carcinogenicity, immunosuppression

## Abstract

Photodynamic Therapy (PDT) is a non-invasive treatment successfully used for neoplastic, inflammatory and infectious skin diseases. One of its strengths is represented by the high safety profile, even in elderly and/or immuno-depressed subjects. PDT, however, may induce early and late onset side effects. Erythema, pain, burns, edema, itching, desquamation, and pustular formation, often in association with each other, are frequently observed in course of exposure to the light source and in the hours/days immediately after the therapy. In particular, pain is a clinically relevant short-term complication that also reduces long-term patient satisfaction. Rare complications are urticaria, contact dermatitis at the site of application of the photosensitizer, and erosive pustular dermatosis. Debated is the relationship between PDT and carcinogenesis: the eruptive appearance of squamous cell carcinoma (SCC) in previously treated areas has been correlated to a condition of local and/or systemic immunosuppression or to the selection of PDT-resistant SCC. Here we review the literature, with particular emphasis to the pathogenic hypotheses underlying these observations.

## 1. Introduction

Photodynamic therapy (PDT) can be done by topically applying the photosensitizer prodrug 5-aminolaevulinic acid (ALA) or its methylated ester (MAL), which are converted by the haem biosynthetic pathway predominantly to protoporphyrin IX (PpIX). Subsequent activation by light of an appropriate wavelength produces reactive oxygen species (ROS), especially singlet oxygen, triggering both apoptosis and necrosis of target cells. PDT is a well-established treatment for actinic keratosis (AK), in situ squamous cell carcinoma (Bowen’s disease), superficial and nodular basal cell carcinoma (BCC) [1]. Experimental and clinical studies also demonstrated various anti-inflammatory effects and immunological activities of PDT [2,3,4,5]. The goal of Non Melanoma Skin Cancer (NMSC) treatment with PDT is to achieve complete eradication of the tumor while preserving aesthetically and functionally important structures. PDT displays several major strengths: it is a non-invasive, easily repeatable, outpatient treatment that can be applied to wide areas of affected skin with an overall good profile of safety. PDT can be used in elderly patients in whom surgery is contraindicated, in immuno-depressed subjects, or to treat large or multiple lesions localized in poorly healing areas, i.e., lower legs. Moreover, PDT shows superior cosmetic outcome compared with surgery and cryotherapy, with no scarring and pigmentary changes [6,7]. Faced with the above listed advantages, PDT is not without burden, with side effects that can be classified, according to the time of onset, in early (immediately or within days after treatment) and late (after weeks or months) onset side effects.

## 2. Early Onset Side Effects

Pain and Local Skin Reactions (LSRs), including erythema, edema, desquamation, or pustulae, often in association with each other, are commonly observed in course of exposure to the light source and in the hours/days immediately after PDT. More rarely, urticaria, contact dermatitis, or erosive pustular dermatosis of the scalp (EPDS) occur. PDT has also a significant effect on the immune system, with acute onset, but with a potential long-term effect on treatment-related carcinogenesis.

### 2.1. Pain

Pain is an issue of general concern, as it represents the most frequent and limiting side effect of conventional PDT, with up to 58% of patients reporting severe pain [8]. Painful burning sensation usually starts immediately or very early during light exposure, becoming rapidly very intense, with a peak in the first minutes of treatment. Thereafter, pain usually tends to decrease or even subside towards the end of the treatment [9]. In some cases, pain can be so severe to induce the premature stop of light exposure, which results in insufficient PpIX formation and inadequate therapeutic result. Pain can also induce systemic symptoms. Borroni et al. reported the occurrence of post-MAL-PDT acute postoperative hypertension in 8 out of 36 patients (22%); 11% of patients developed hypertensive crisis, requiring immediate treatment [10]. Interestingly, the majority of these patients had a positive history for hypertension, which may represent an important risk factor and identify high-risk subjects [10]. PDT has a significant temporary impact on patients’ quality of life, with a marked increase of the dermatology life quality index (DLQI) scores from 1.6 ± 1.7 prior to PDT to 7.3 ± 4.9 immediately post PDT [11]. Moreover, most patients experiencing severe pain are dissatisfied about effectiveness, convenience and overall experience. Pain can negatively influence patients’ adherence, leading to refusal of further treatment [12].

The main bias about pain is the subjective evaluation of this individual sensation, with large inter-patient variation. The commonest score method is the visual analogue scale (VAS), with pain scores categorized into mild (0–40), moderate (41–70) and severe (71–100). Unfortunately, VAS is arbitrary, with not reproducible results. Assessing pain by measuring time for pain induction, as proposed by Mikolajewska et al., may be less biased, providing a reliable source of information for comparisons [13]. The exact mechanism of PDT-related pain is yet unknown. Reactive oxygen species (ROS) are the main mediators of pain experience during PDT, and contribute to stimulation of sensory neurons that conduct pain to sensory cortex of the brain. Intensity of pain can be determined by the depth of singlet oxygen production in the skin, which in turn depends on the nature of the photosensitizer and on the wavelength of the stimulating light. Local hypoxia secondary to oxygen-consuming reactions, like PpIX photobleaching or tumour destruction, can cause a decrease of the pH in the tissue, and trigger pain signals due to the low oxygen level around the mitochondria-rich nerve endings [14,15]. Pain is the result of the interplay between many intrinsic and extrinsic factors. No correlation was found with age or sex, and no studies investigated racial influence [16,17,18]. Skin phototype seems to not influence pain experience, although some studies reported higher intensity of pain in fair-skinned patients. However, these patients are constitutionally more prone to develop sun-induced tumors in larger areas than dark phototypes [17,18,19].

About photosensitizer, many studies compared the pain intensity experienced using ALA or MAL. Unfortunately, these comparisons are difficult to interpret, as clinicians use the drugs differently in clinical practice; moreover, some authors compared branded versus compounded drugs. Kasche et al. [15] evaluated 69 patients affected by multiple AKs on the scalp, and reported that ALA-PDT caused a higher level of pain than MAL-PDT. Similar results were obtained some years later by Steinbauer et al. [20]. Gaal et al. [21] compared the pain caused by ALA-PDT and MAL-PDT in different body areas (head, trunk, extremities), and found that ALA-PDT was more painful than MAL-PDT in all cases, but the difference was statistically significant only for head lesions.

On the other hand, Ibbotson et al., in a cohort of patients affected by Bowen’s disease and BCC, found no significant differences in VAS scores between ALA-PDT and MAL-PDT [22]. Such results were confirmed by Yazdanyar et al., who reported no significant difference in an intra-individual split-forehead and scalp study, where MAL-PDT and ALA-PDT were given to each patient in two similar areas [23]. Some studies used measurements of fluorescence intensity to evaluate PpIX generation with both photosensitizers. Pretreatment fluorescence directly correlates with pain intensity and is a good predictor of erythema and lesion clearance [24,25,26,27,28]. The redness of the actinic lesions was found to be related to PDT-induced pain, the reduction of actinic area, and the cure rate. The redder the actinic area, the better the treatment outcome and the more pain experienced [29]. It is not clear, however, whether different incubation times may influence PpIX concentration and clinical outcome.

A few studies investigated the correlation between clearance rate and different incubation times with both ALA and MAL, showing no significant differences between 1 h vs. 3 h regimens [30,31]. Lerche et al. recently introduced the concept of pulse–PDT, in which MAL is applied for 30 min under occlusion before it is removed [32]. After removal, the skin is covered with a light-impermeable dressing for 2.5 h, followed by red light illumination. The short-time incubation should promote selective PpIX accumulation in the mitochondria and the endoplasmic reticulum (which are considered the main targets for achieving apoptosis), preventing excessive PpIX production in the surrounding tissue. This would limit death to diseased cells and decrease the severity of adverse events such as pain and erythema, with no influence on treatment efficacy [32,33]. Fluence shows a strong positive correlation with pain, lower fluences being less painful [28,34,35,36,37]. Light dose also plays an important role in modulating pain sensation. Radakovic-Fijan et al. found no significant correlation between high light dose and pain intensity when total light dosage was higher than 70 J/cm^2^ [38]. Consistent with these observations, Wang et al. proposed a threshold theory for PDT-induced pain, postulating a positive correlation with both fluence rate and dose below a certain threshold (rate of 60 mW/cm^2^, dose of 50 J/cm^2^) [39]. Exceeding this threshold, no significant increase in pain is experienced [39]. The link may be ROS generation. Increasing light dose and fluence rate causes a progressive increase in ROS production; when the threshold is reached, desensitization of nociceptors and/or saturation of cell capacity to produce ROS may determine a plateau of pain perception. The constant and slow dynamics of ROS production is probably the mechanism through which Daylight PDT (DL-PDT) is quite less painful respect to conventional PDT, with increased patient tolerance and satisfaction. Compared to conventional PDT, pain intensity during DL-PDT is significantly lower, probably due to continuous production and photoactivation of small amounts of PpIX, with decreased local concentration of ROS and, consequently, reduced stimulation of nerve endings [40,41]. Other important factors influencing pain are lesion type, location and treatment area size. Many studies identified AK as the most painful lesion to treat, with head and neck location having the greatest impact on pain perception, because of the high nerve density; lesions located on the limbs cause a greater degree of pain than those on the trunk [11,17,18,19,42]. Nevertheless, other researchers found nodular BCC and BD to be the most painful lesions to treat, suggesting a role for lesion thickness [16,24]. The treatment area size positively correlates with severe pain, with larger areas being more painful [12,17,18]. The first treatment is frequently less painful than the second, as demonstrated by Lindeburg et al. [43], a patient with low pain experience during the first PDT has a greater risk of more pain during the second PDT, while a patient with high pain experience during the first PDT is more likely to feel a reduction of pain during the following light exposure [43].

Pain management is a major challenge. Different strategies, including cold air analgesia, topical anesthesia, infiltration anesthesia, nerve block, hypnosis, have been studied, none of them being completely effective [44,45,46]. When indicated, DL-PDT is the real painless alternative to conventional PDT.

### 2.2. LSRs

**Figure 1:**
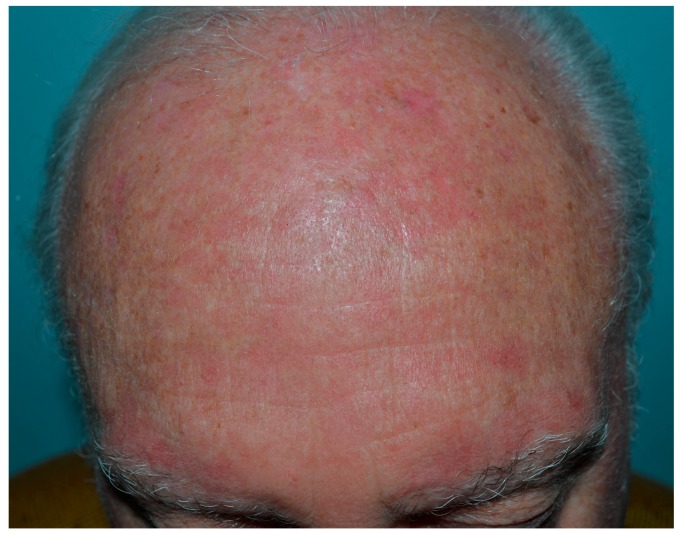
Intense erythema twenty-four hours after PDT.

**Figure 2:**
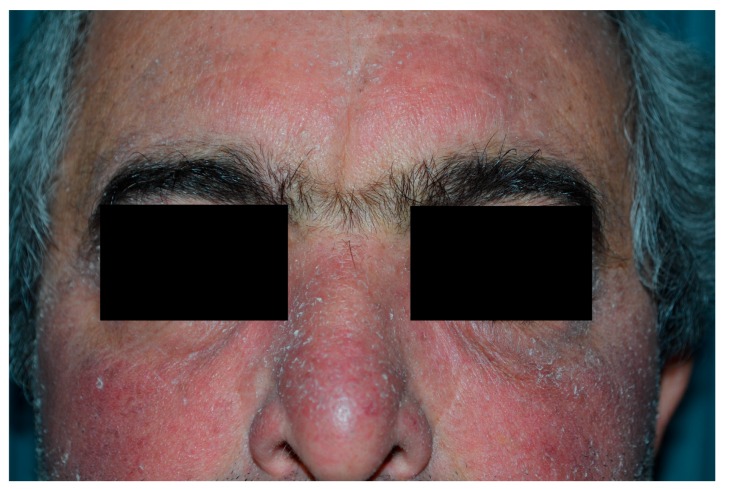
Diffuse scaling seven days after PDT.

**Figure 3:**
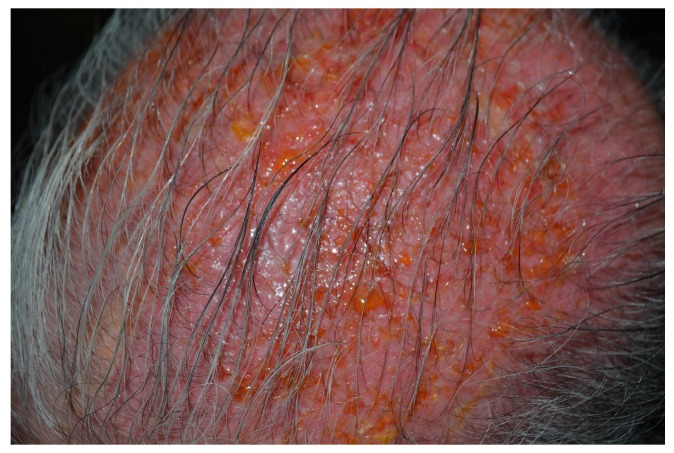
Erythema, vesicles and erosions seventy-two hours after PDT.

Erythema (Figure 1) and edema are the main phototoxic effects of PDT and develop in the treated area during and after light exposure [47]. The often severe erythema can be followed by crusting and generally resolves in 1–2 weeks (Figure 2). In one large study of patients (*n* = 2031) receiving topical PDT over a 5-year period, erythema and oedema occurred in 89% of subjects and 80% reported scaling and itch. Crusting (9%), pustules (6%), erosions (1.2%) and infections (0.4%) were other reported adverse effects (Figure 3) [48]. Especially during PDT treatment of large areas on the face and scalp, patients are discomforted by the inflamed appearance that may prevent them from going to work for days [49].

Brooke and colleagues studied the effects of ALA-PDT on human skin, demonstrating that the acute inflammatory response comprises immediate stinging, followed by a more prolonged erythema, and that histamine, at least in part, mediates the acute reaction to PDT. Post-treatment dermal histamine levels peak at 30 min after light exposure, remain stably elevated for 4 h, and gradually return to baseline by 24 h posttreatment [50]. However, a recent clinical trial which evaluated the impact of oral H1 antihistamine therapy in the reduction of LSR showed no effects both on inflammatory response and ALA-PDT efficacy [51]. If histamine is a key mediator of the immediate urticarial response, the delayed erythema is more closely attributable to other proinflammatory mediators such as prostaglandin E2 and nitric oxide, owing to their vasodilatory properties and their involvement in apoptosis and tumorigenesis in experimental models [52]. However, these data refer to human healthy skin, and may not reflect the changes induced by PDT in damaged skin.

There are not many studies on the inflammatory effects of PDT performed using photosensitizers at lower concentrations than usual. Wiegell et al. [53] compared the erythema resulting from PDT with MAL 16% (standard) and MAL 8%, finding no significant difference. More recently, Fabricius et al. [54] evaluated four different MAL concentrations (16%, 2%, 0.75%, 0.25%) on 24 healthy volunteers, showing that use of lower concentrations was strongly correlated with lower PpIX fluorescence and less intense erythema. It was postulated that cell death at lower MAL concentrations could occur by apoptosis more than necrosis, thus reducing the amount of inflammation.

The same authors studied the correlation between treatment time, photobleaching, inflammation and pain after MAL-PDT on tape-stripped skin in healthy volunteers [32]. The results showed a significant correlation between incubation time, time until illumination and photobleaching, with a positive correlation between photobleaching and erythema. Shorter incubation time and shorter time until illumination also result in less pain, confirming the correlation between pain score and erythema evidenced by Piffaretti and Barge [26,37]. As for pain intensity, lower severity of local adverse events after DL-PDT than after c-PDT was reported in an intra-patient, right/left study in patients affected by AK of the forehead [41], showing that continuous production and photoactivation of small amounts of PpIX is an effective way to control inflammation. Application of superpotent corticosteroid before and just after PDT reduced erythema 24 h after treatment of multiple AKs on the face and scalp, with no influence on the efficacy of the treatment, thus making PDT treatment of large visible areas more acceptable and reducing down time. The effect on mediators of inflammation, but not histamine, may explain the lack of efficacy of this approach on immediate post-treatment erythema and oedema [55]. Also, light protection after PDT with inorganic sunscreens demonstrated an erythema-reducing effect [56].

### 2.3. Urticaria

Urticarial reactions to PDT with ALA and MAL were described in the literature [57,58]. A 2008 study reported a 0.9% prevalence (12/1353 patients) for severe itching and wheals within the first minute of illumination [59]. The patients most predisposed to reaction were those who had received more than 7 courses of treatment (3.8% prevalence). The proposed mechanism was histamine release from mast cells in the dermis. This pathogenesis is consistent with the recurrent nature of the reactions in subsequent treatments, the satisfactory control of these reactions through administration of an antihistamine, such as cetirizine, before treatment, and the immediate appearance of urticaria in areas not previously treated with PDT. Only two pediatric cases of urticaria during PDT were reported until now [60].

### 2.4. Contact Dermatitis

Allergic contact dermatitis was reported with MAL and, more rarely, with ALA [61,62,63,64,65,66]. Despite their structural similarity, no evidence of cross-reactivity between the two agents was highlighted until now. In the twenty cases reviewed by Pastor-Nieto et al., patch tests with the licensed 16% MAL preparation were positive in all patients while patch tests with the vehicle were negative, confirming the causative role of the active ingredient [67]. A single case of systemic allergic contact dermatitis caused by MAL was recently described in a patient with keratosis–ichthyosis–deafness syndrome [68]. It is likely that the incidence of sensitization to MAL is underestimated, as a number of intense inflammatory post-PDT reactions probably reflects genuine contact dermatitis. Conversely, the use of many different ALA compounded drugs could explain the lower reported occurrence of contact dermatitis caused by this molecule.

### 2.5. Immunosuppression

PDT has a significant effect on the immune system, by either stimulating or, in some circumstances, repressing innate and adaptive immune response [69]. PDT causes release or expression of various pro-inflammatory and acute phase response mediators from the treated site, with local recruitment of neutrophils and other inflammatory cells in large numbers and activation of the complement system, targeting the tumor microenvironment [70,71,72]. In addition to stimulating local inflammation, PDT can induce potent, systemic, antigen specific anti-tumor immunity [73]. The other side of the coin is the ability of PDT to cause local and systemic immunosuppression, with reduction of delayed-type hypersensitivity (DTH) responses to recall antigens. By measuring Mantoux erythema and diameter, Matthews and Damian found that, at the light doses and irradiance rates in current clinical use, both ALA-PDT and MAL-PDT were locally immune suppressive even after one treatment session [74]. Moreover, it was shown that PDT reduces the number of Langerhans cells (epidermis-resident antigen-presenting cells) both in healthy skin and in biopsy samples from BCC. Such reduction can induce antigenic tolerance and can be responsible for suppression of contact hypersensitivity both at the site of irradiation (local immunosuppression) as well as at distant, non-irradiated sites (systemic immunosuppression), with potential negative impact on antitumor response [75,76]. In this respect, Thanos et al. showed that both oral and topical nicotinamide (vitamin B3) reduce the immune suppressive effects of PDT on DTH responses in humans, and proposed administration of nicotinamide as a simple method to increase the effectiveness of PDT [77]. Moreover, the same authors demonstrated a synergic effect of administration of nicotinamide and low irradiance rate PDT, with no negative impact on tumour clearance rate [78]. On the basis of these considerations, we could consider the association of low light intensity DL-PDT and oral nicotinamide as the best strategy to achieve good therapeutic efficacy with high safety profile. The exact mechanism by which nicotinamide exerts this effect is still not fully understood. Probably is involved its ability, as an NAD precursor, to replenish cellular ATP levels decreased by PDT, thus favoring the highly energy-dependent processes of DNA repair. Furthermore, nicotinamide does not exert antioxidant effects in vitro in human keratinocytes, and, consequently, does not decrease ROS generation, which is required for the antitumoral action of PDT [77,79].

### 2.6. Miscellanea

It is known that PDT exerts a wide spectrum of antimicrobial effects, so it is not unexpected that occurrence of infections at the site of treatment is a rare complication, with only four cases of bacterial cellulitis reported over 700 treatments [80]. To date, only one case of EPDS was reported, with extensive sterile pustular lesions, non-healing erosions, and crusting of the scalp [81]. Interestingly, three cases of EPDS were recently reported after ingenol mebutate application, suggesting that post-treatment inflammation could act as trigger factor in highly photodamaged areas [82,83]. The good response to both topical and systemic steroids could lead us to hypothesize a common pathogenic mechanism linked to neutrophilic infiltration, which is a key component of the inflammatory response in both treatments [55,84]. Gemigniani et al. reported a case of complete left peripheral facial palsy occurred 1 week after topical PDT for left hemifacial actinic keratosis [85]. The authors considered it as a possible, although uncommon, complication of PDT, on the basis of the close relation among the treated zone, the superficial localization of facial nerve branches, and the short elapsed time.

## 3. Late Onset Side Effects

### 3.1. Pigmentary Changes and Scarring

PDT can rarely induce hyperpigmentation and scarring. In the large experience of the Scottish PDT Centre, pigmentary change and mild/moderate scarring accounted only for 1% and 0.8% of lesions treated, respectively, on a patient population predominantly of skin phototypes I–III [86]. Hyperpigmentation is generally transient, with slow resolution within the months following PDT. Hypopigmentation, presumably due to phototoxic damage to melanocytes, can also occur, although this is not well documented in the literature [47].

### 3.2. Bullous Pemphigoid

Two cases of post-PDT bullous pemphigoid (BP) were described, one strictly confined to the areas treated with PDT for Bowen’s disease, the other involving other sites too [87,88]. In both cases, BP lesions were detected after 3–4 months, at follow-up visit. The pathogenetic mechanism remains unknown; Wolf’s isotopic response was suggested as possible explanation.

### 3.3. Carcinogenicity

In the spectrum of the possible side effects caused by PDT, the most worrisome is certainly its potential to induce or stimulate skin carcinogenesis. Several reports showed onset of BCC, keratoacanthoma and invasive SCC after treatment with PDT [89,90,91,92,93,94,95,96,97]. In 1997, a case of melanoma of the scalp, at a site repeatedly exposed to topical ALA-PDT for solar keratoses and superficial SCCs, was reported by Wolf et al. [98]. In 2009, Schreml and colleagues described another case of melanoma developed after PDT treatment of BD on the right cheek [99]. The cases are summarized in Table 1. These reports highlight the dilemma of whether PDT may promote tumor development and growth, and should be considered with caution. Indeed, these patients often have a great predisposition to skin cancer (immunosuppression, prior history of NMSC, heavily photodamaged skin, multiple treatment fields) and PDT could have a coincidental, rather than causal, role in promoting carcinogenesis. The carcinogenic risk may be the consequence of different pathogenic mechanisms, including the previously discussed immunosuppression, mutagenesis and isotopic response. The mutagenic effect of PDT is controversial. Some authors affirm that PDT is not directly mutagenic on DNA, others demonstrated that ROS generated after PDT photosensitization can cause DNA damage and oncogene activation [100,101]. Kick et al. described the PDT-related induction of some proto-oncogenes (c-jun and c-fos) involved in the carcinogenesis of human epithelial cells [102]. Giri et al. reported a particular effect of the photosensitizer protoporphyrin in mouse skin (double dose-dependent effect), resulting in the in situ generation of ROS, and able to induce DNA damage in normal epithelial cells [103]. They demonstrated that mice treated by PDT face an anti-tumoral effect (destruction of tumor cells) with a high dose of haematoporphyrin (5 mg/kg) and a pro-tumoral result (DNA damage) with a lower dose (2.5 mg/kg) [103]. Miyazu and colleagues evaluated telomerase protein expression in noncancerous bronchial epithelium of patients with lung cancer, and concluded that PDT is useful to treat lung cancer, but does not destroy normal cells that express telomerase and are, for this reason, predisposed to SCC development [104]. This aspect turns out to be relevant when the important role of telomerase is considered in skin carcinogenesis [104]. PDT could also modify the course of tumor. Gilaberte and colleagues studied recurrences and aggressiveness of skin tumors non respondent to PDT, reporting an increased Epidermal Growth Factor Receptor (EGFR) expression after MAL-PDT [105]. Some authors found high expression of EGFR in tumors characterized by aggressiveness, poor prognosis, short survival of patients and development of resistence to cytotoxic agents [106]. This correlation was demonstrated also in SCC [107]. Gilaberte and colleagues also focused on the role of mitogen-activated protein kinase (MAPK), mediated in most human cancer by fosforilation of ERK1/2. They hypothesized that PDT may promote the selection of more aggressive tumor cells, and the MAPK/ERK signal pathway may be involved in the resistance to PDT [105]. Moreover, the activation of EGFR induces stimulation of ERK, with consequent overexpression of cyclin D1, which is frequently involved in keratinocyte carcinogenesis [108]. In this regard, Moreno Romero et al. reported two cases of rapidly growing squamous cell carcinoma after treatment of ingenol mebutate for AKs on the forehead (time to onset: 4 weeks) and the neck (time to onset: 5 weeks), and proposed that, in some cases, the inflammatory process induced by ingenol mebutate could accelerate the transformation of AKs into SCCs [109]. It is interesting to highlight that the MAPK/ERK signal pathway is involved also in the mechanism of action of ingenol mebutate and could explain, at least in part, this paradoxical response, which consists of reduction of cell viability and proliferation, and, on the other hand, promotion of tumor cell growth [110].

Lastly, the development of skin cancer at PDT-exposed sites could be explained by the concept of immunocompromised district (ICD), introduced in 2009 by Ruocco and colleagues [111]. An ICD is a skin-damaged area with regional imbalance of the immune response, vulnerable to a secondary distinct disease, including skin tumors. Trauma-related to light exposure, as well as previous unsuccessful treatments (both physical and chemical) in the same area, frequently used in combination in patients with NMSC, might render the PDT-field a *locus minoris resistentiae*, which means a site of the body with lesser resistance than the rest of the body to the development of disease, due to a localized immune dysregulation [112]. In this respect, Ratour-Bigot et al., in their series of 105 patients treated with PDT for BD, hypothesized a potential direct relationship between PDT and the development of SCC only when SCC appears in the PDT field [97].

The role of PDT as promoter of skin malignancies remains not completely understood and its influence on tumor development in humans requires further study. However, taking into account the cases of skin cancers after PDT that were reported in literature, it appears crucial to perform continued and careful follow-up after this useful treatment, especially in patients with multiple risk factors for skin cancers, and to make biopsies when, in a treated area, a new suspicious lesion appears or invasion is likely.

## 4. Conclusions

PDT can be considered an effective and safe treatment options for NMSC. Pain is the most clinically relevant short-term complication, that also reduces long-term patient satisfaction. Simpler and more tolerable treatment procedures (DL-PDT, pulse-PDT), employing lower fluences and light doses, seem to give significant results in term of pain reduction with no influence on clinical response rate. A closer look to PDT-related carcinogenesis is mandatory. A greater understanding is needed of how PDT might induce skin tumorigenesis, to trace the contours of such paradoxical phenomenon. Considering the long time lapse (up to 7 months) between PDT and tumor onset, it is not an hazard to hypothesize that the real incidence of PDT-related carcinogenesis may be higher than that reported until now, probably because of misdiagnosis or underreport. The case series reported in literature are retrospective, involving small cohort of patients; prospective studies involving a larger number of patients, with frequent and prolonged follow-up, may be helpful to not miss PDT-induced SCC. Moreover, in the context of occurrence of skin cancer after treatment, comparative studies between PDT and other field-directed therapies, i.e., diclofenac, ingenol mebutate and imiquimod, are requested, in order to identify any possible risk factor for therapy-promoted carcinogenesis.

## Figures and Tables

**Table 1:** Summary of the reported cases of skin cancers after photodynamic therapy.

Reference	Patients Treated	Lesions Treated	Topical Photosensitizer	Site of Treatment	Sessions	Time between PDT and Onset of Skin Cancer	Type of Skin Cancer Observed during Follow-Up after PDT
Case series							
Bardazzi et al. (2015) [94]	357	AKs	ALA	Face, scalp, chest and hands	1 for 10 patients; 2 for 3 patients; 3 for 4 patients ^3^	7 months (mean time) ^3^	17 Invasive SCCs + 7 BCCs and BD
Ratour-Bigot et al. (2016) [97]	105	BD	MAL	Head, neck, limbs, trunk	4 (mean)	6 months (mean time)	16 Invasive SCCs
De Graaf et al. (2006) [90]	40	None ^1^	ALA	Forearm and hand	Not specified	During a two years follow-up	15 invasive SCC in 9 patients
Calista (2014) [92]	15	AKs	MAL	Scalp, forehead and ears	2	6 months	5 Invasive SCCs
Case studies							
Liang et al. (2014) [93]	2	BD	MAL	Right temple, chest	2	2 months (right temple), 4 months (chest)	Invasive SCC
Wolf et al. (1997) [98]	1	Solar keratoses and SCCs	ALA	Scalp	7	6 months	Melanoma (Clark level II; Breslow index 0.4 mm)
Varma et al. (2000) [89]	1	EQ	ALA	Glans penis	4	4 months	Invasive SCC
Maydan et al. (2006) [91]	1	AKs	ALA	Face	N/A	N/A	Keratoacanthoma
Schreml (2009) [99]	1	BD	Not reported	Right check	1	2 months	Melanoma (Clark level III; Breslow index 0.4 mm)
Ibbotson et al. (2011) [43]	1	BD	Not reported	Periocular	Not reported	Short interval, not better defined	Invasive SCC
Gogia et al. (2013) [95]	1	AKs	ALA ^2^	Face	1	3 weeks	Eruptive keratoacanthomas
Ramirez et al. (2015) [96]	1	AKs	ALA	Forearms	Not reported	3 weeks	Eruptive keratoacanthomas

^1^ Preventive use in transplant recipients; ^2^ With microdermoabrasion; ^3^ Number of sessions and time between PDT an onset of skin cancer not specified for BCCs and BDs. PDT = photodynamic therapy; SCC = squamous cell carcinoma; AKs = actinic keratosis; ALA = aminolevulinic acid; MAL = methyl aminolevulinate; BCC = basal cell carcinoma.
